# A triple drug combination targeting components of the nutrient-sensing network maximizes longevity

**DOI:** 10.1073/pnas.1913212116

**Published:** 2019-09-30

**Authors:** Jorge Iván Castillo-Quan, Luke S. Tain, Kerri J. Kinghorn, Li Li, Sebastian Grönke, Yvonne Hinze, T. Keith Blackwell, Ivana Bjedov, Linda Partridge

**Affiliations:** ^a^Institute of Healthy Ageing, Department of Genetics, Evolution and Environment, University College London, WC1E 6BT London, United Kingdom;; ^b^Section on Islet Cell & Regenerative Biology, Joslin Diabetes Center, Boston, MA 02215;; ^c^Department of Genetics, Harvard Medical School, Boston, MA 02115;; ^d^Department of Biological Mechanisms of Ageing, Max Planck Institute for Biology of Ageing, D-50931 Cologne, Germany;; ^e^Department of Molecular Neuroscience, Institute of Neurology, WC1N 3BG London, United Kingdom;; ^f^Department of Cancer Biology, Cancer Institute, University College London, WC1E 6DD London, United Kingdom

**Keywords:** aging, polypharmacology, trametinib, rapamycin, lithium

## Abstract

Increasing life expectancy is causing the prevalence of age-related diseases to rise, and there is an urgent need for new strategies to improve health at older ages. Reduced activity of insulin/insulin-like growth factor signaling (IIS) and mechanistic target of rapamycin (mTOR) nutrient-sensing signaling network can extend lifespan and improve health during aging in diverse organisms. However, the extensive feedback in this network and adverse side effects of inhibition imply that simultaneous targeting of specific effectors in the network may most effectively combat the effects of aging. We show that the mitogen-activated protein kinase kinase (MEK) inhibitor trametinib, the mTOR complex 1 (mTORC1) inhibitor rapamycin, and the glycogen synthase kinase-3 (GSK-3) inhibitor lithium act additively to increase longevity in *Drosophila*. Remarkably, the triple drug combination increased lifespan by 48%. Furthermore, the combination of lithium with rapamycin cancelled the latter’s effects on lipid metabolism. In conclusion, a polypharmacology approach of combining established, prolongevity drug inhibitors of specific nodes may be the most effective way to target the nutrient-sensing network to improve late-life health.

Aging is a complex process of progressive cell, tissue, and systemic dysfunction that is involved in the etiology of age-related diseases (1). Genetic, dietary, and pharmacological interventions can ameliorate the effects of aging in laboratory animals and may lead to therapies against age-related diseases in humans (2–4).

In organisms ranging from invertebrates to mammals, reducing the activity of the nutrient-sensing mechanistic target of rapamycin (mTOR) and insulin/insulin-like growth factor signaling (IIS) network can promote longevity and health during aging (2, 3). Lowering network activity can also protect against the pathology associated with genetic models of age-related diseases (1, 2). The network contains many drug targets, including mTOR, mitogen-activated protein kinase kinase (MEK), and glycogen synthase kinase-3 (GSK-3) (Fig. 1*A*). Down-regulation of mTOR activity by rapamycin, GSK-3 by lithium, or MEK by trametinib can each individually extend lifespan in laboratory organisms (5–11), and brief inhibition of mTOR has recently been shown to increase the response of elderly people to immunization against influenza (12). In addition, both mTOR and MEK inhibitors have been shown to reduce senescent phenotypes in human cells (13), while increasing concentrations of lithium levels in drinking water correlate with reduced all-cause mortality in a Japanese population (10). An advantage of pharmacological interventions is that the timing and dose of drug administration are relatively simple to optimize, and drugs can be easily combined (4, 14–16). Combination drug treatments also have the potential to counter resistance from feedback and to reduce each other’s side effects (17). Rapamycin, trametinib, and lithium each target different kinases and transcription factors to extend lifespan (5, 8, 11), and therefore their effector mechanisms are at least partially different from each other. Simultaneous inhibition of multiple targets within the nutrient-sensing network may hence be needed to optimize effector outputs and health benefits. Here, we measure the effects of combination treatments of rapamycin, lithium, and trametinib on lifespan and other traits, using *Drosophila* as a model organism.

**Fig. 1.**
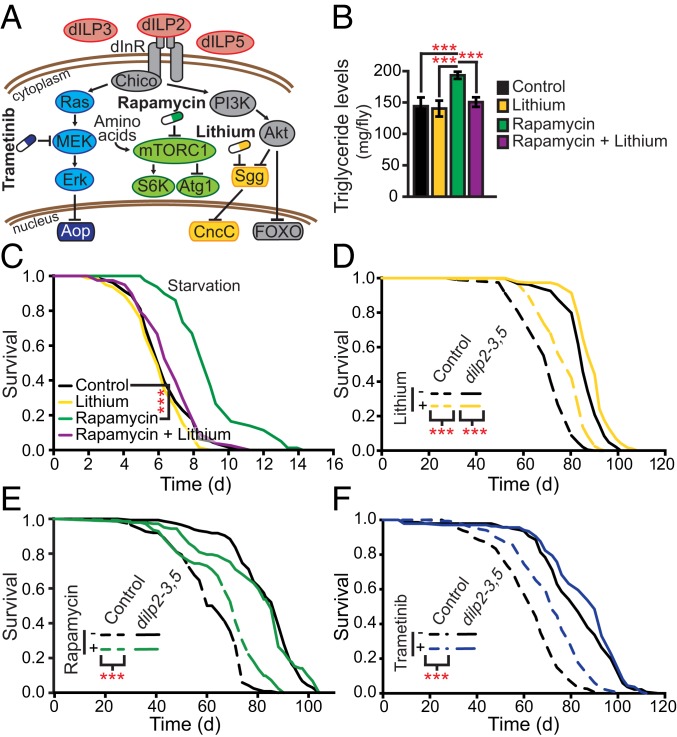
Lithium blocks negative side effects of mTORC1 and IIS inhibition. (*A*) A simplified diagram of the *Drosophila* nutrient-sensing network showing the target kinases of rapamycin, trametinib, and lithium. Lithium reversed the (*B*) hypertriglyceridemia (*n* = 6 replicas of 5 flies per condition, 1-way ANOVA) and (*C*) starvation resistance induced by rapamycin (50 µM) (*n* = 75). (*D*) Lithium treatment significantly extended lifespan of both *w*^*Dah*^ and *dilp2-3,5* mutant flies. Neither (*E*) rapamycin (*P* = 0.58) nor (*F*) trametinib (*P* = 0.14) further extended lifespan of *dilp2-3,5* mutant flies [log-rank test (*n* = 150)]. Cox Proportional Hazard analysis showed a significant genotype by treatment interaction for rapamycin (*P* = 0.002) and trametinib (*P* = 0.0018). Error bars represent SEM. ***P < 0.001 (1-way ANOVA or log-rank test).

## Results and Discussion

Rapamycin treatment, from *Caenorhabditis elegans* to humans, is associated with altered metabolism, including hypertriglyceridemia and obesity (5, 18). Alone, a lifespan-extending dose of lithium (11) did not alter triglyceride levels, but simultaneous treatment with both lithium and rapamycin reversed the dyslipidemia caused by rapamycin (Fig. 1*B*). To confirm that this change in lipid levels was physiologically relevant, we pretreated (14 d) flies with lithium, rapamycin, or a combination, and assessed their survival under starvation. Lithium did not alter survival under starvation conditions, while rapamycin increased it (Fig. 1*C*). Consistent with their effects on lipid levels, combining lithium and rapamycin treatment resulted in control levels of starvation resistance (Fig. 1*C*). Lithium can therefore reverse metabolic storage alterations associated with mTOR inhibition.

Lithium inhibits GSK-3 activity to extend lifespan (11), implying that activation of GSK3 is likely, if anything, to shorten lifespan. Inhibition of IIS in the canonical PI3K pathway can extend lifespan and health span, but reduces inhibitory phosphorylation of GSK3 by Akt (Fig. 1*A*), and hence activates GSK3 (4), a potentially deleterious side effect of lowered IIS (19). We therefore tested whether lithium could have additive effects in combination with genetic inhibition of IIS upstream of Akt. Lithium was able to further extend the lifespan of flies lacking the insulin-like peptides 2, 3, and 5 (*dilp2-3,5*) (Fig. 1*D*) (20). In contrast, rapamycin or trametinib, neither of which inhibit GSK3, were not able to extend the lifespan of *dilp2-3,5* flies (Fig. 1 *E* and *F*). Lithium thus reverses an adverse side effect of inhibition of the canonical IIS pathway.

**Fig. 2.**
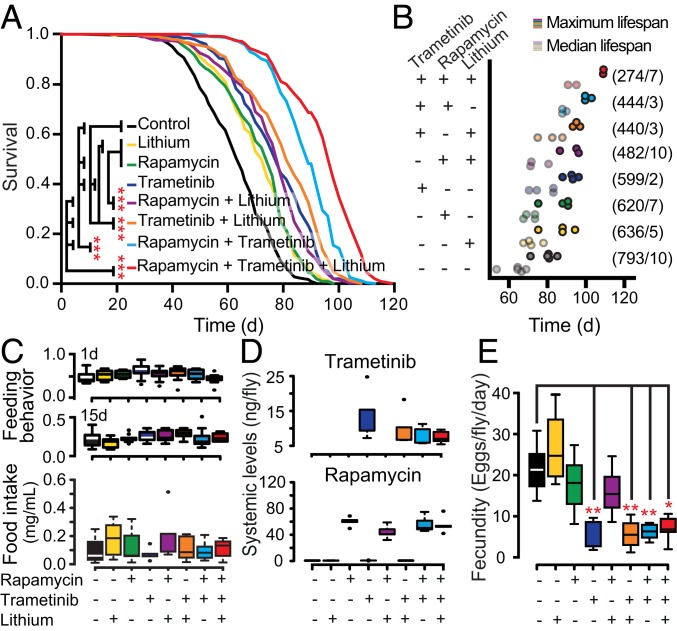
A triple drug combination maximizes longevity. (*A*) Representative survival curve and associated pairwise log-rank tests. (*B*) Replicated median/maximum lifespans plotted for all single (*n* = 4), double (*n* = 3), and triple (*n* = 2) combinations of rapamycin, trametinib, and lithium treatments. Each lifespan contained 130 to 200 flies per treatment. Numbers in parentheses show (total number of flies/number of censors). (*C*) Proboscis extension feeding behavior assay (1 and 15 d of treatment; *Top* and *Middle*) and quantification of ingested nonabsorbable (*Bottom*) blue dye (*n* = 8 replicas of 4 to 5 flies 15 d old, 1-way ANOVA with Dunnett’s test). (*D*) Mass spectrometry of systemic trametinib (*Top*) or rapamycin (*Bottom*) levels when other drugs were coadministered (*n* = 5, 1-way ANOVA). (*E*) Fecundity of treated (15 d) flies within a 24-h period (*n* = 8 replicas of 4 to 5 flies). Error bars show Tukey whiskers, and outlying data points are shown as dots. *P < 0.05, **P < 0.01, ***P < 0.001 (Kruskal−Wallis test and Dunn’s pairwise tests).

Because rapamycin, lithium, and trametinib extend lifespan by at least partially independent mechanisms, we investigated the effects on lifespan of their double and triple combinations. Double combinations of lithium and rapamycin, lithium and trametinib, or rapamycin and trametinib produced a reproducibly greater lifespan extension than controls, on average 30%, compared to each compound alone, which extended lifespan by an average of 11% (Fig. 2 *A* and *B* and Dataset S1). Importantly, the triple combination of rapamycin, trametinib, and lithium promoted longevity beyond that of the double combinations, extending median lifespan by 48% (Fig. 2 *A* and *B* and Dataset S1). Thus, each compound independently displayed an additive effect on lifespan. The additive effect of rapamycin, trametinib, and lithium on lifespan is unlikely to have been due to changes in feeding behavior, because feeding frequency, food intake, and drug uptake were unaltered by the treatment regimens (Fig. 2 *C* and *D*). Fecundity is often reduced in interventions that promote lifespan extension (21), and this could provide a potential explanation for the greater longevity with drug combinations. However, at the concentrations used, only trametinib and combinations containing trametinib significantly reduced fecundity (Fig. 2*E*). Importantly, the triple drug combination did not reduce egg laying below that achieved with double trametinib-containing combinations, or trametinib treatment alone (Fig. 2*E*). Thus, a trade-off with fecundity is unlikely to explain the greater longevity observed with the triple drug combination.

Given the complex nature of the aging process, it is unlikely that the most effective preventative antiaging therapy could be achieved by a single compound with a single target. We have shown that simultaneous inhibition by 3 components of different nodes in the nutrient-sensing network using a combination of drugs already approved for human use is a viable strategy to maximize animal longevity and to reduce a side effect. Rapamycin treatment results in insulin resistance and dyslipidemia in patients and mice (4, 18, 22), and this disturbance manifests as hypertriglyceridemia in *Drosophila* (5). Lithium reversed this and the starvation resistance associated with rapamycin treatment. Taken together, our results highlight a potential therapeutic avenue to promote longevity, coadministrating compounds that act on different nodes of the nutrient-sensing network, to maximize their beneficial effects while minimizing negative side effects.

## Methods

### Fly Stocks, Husbandry, and Lifespan Analysis.

For all experiments, a wild-type white *Dahomey* (*w*^*Dah*^) stock, or, when noted, *dilp2-3,5* mutant flies (*w*^*Dah*^ backcrossed), were used, and raised as previously described (20). LiCl (Sigma) in ddH_2_O, trametinib (LC laboratories) in dimethyl sulfoxide, and rapamycin (LC laboratories) in 100% ethanol were added to sugar−yeast−agar (SYA) medium to a final concentration of 1 mM, 15.6 μM, and 50 μM, respectively (5, 8, 11). Equivalent volumes and concentrations of vehicle were added to SYA medium for control treatments. Drug treatments were started 2 d posteclosion. Female flies (*n* = 130 to 200, 15 to 20 per vial) were sorted onto SYA medium that was replaced every 2 d to 3 d throughout life. Lifespan raw data are provided as Dataset S1. Starvation assay was performed as previously described (11).

### Food Intake, Fecundity, and Triglyceride Measurements.

Feeding behavior (proboscis extension at 1 and 15 d of treatment) and food intake (quantified by dye-calibrated feeding) (4 to 5 flies per replicate, *n* = 8 to 10) were measured as previously described (23). Fecundity was quantified as number of eggs laid within 24 h (15 d), and triglyceride measurements (5 flies per replicate, *n =* 8) were performed as previously described (5, 11).

### Mass Spectrometry.

Flies (*n* = 5, 15 flies) were treated with drugs (15 d), their digestive system was allowed to void (1 h), they were snap frozen, drugs were extracted as previously described (5), and they were resuspended in 100 µL of acetonitrile/isopropanol 70:30 for measurement with an Acquitiy UPLC I-class System/Xevo TQ-S (Waters) with MassLynx and absolute quantification.

## Supplementary Material

Supplementary File
